# Appendiceal Dieulafoy’s Lesion: A Rare Cause of Lower Gastrointestinal Bleeding

**DOI:** 10.70352/scrj.cr.25-0764

**Published:** 2026-04-07

**Authors:** Natsuki Hoshino, Jun Yamamoto, Shogo Takei, Yasuhiro Shimizu, Yusaku Tanaka, Taichi Yabuno, Hiroyuki Hayashi, Yasuhisa Mochizuki

**Affiliations:** 1 Department of Gastrointestinal Surgery, Yokohama Municipal Citizens Hospital, Yokohama, Kanagawa, Japan; 2 Department of Pathology, Yokohama Municipal Citizens Hospital, Yokohama, Kanagawa, Japan

**Keywords:** appendiceal bleeding, Dieulafoy’s lesion, lower gastrointestinal bleeding, appendectomy

## Abstract

**INTRODUCTION:**

Acute lower gastrointestinal bleeding is common in clinical practice. However, it is rarely caused by the appendix, and cases caused by a Dieulafoy’s lesion are even more uncommon.

**CASE PRESENTATION:**

A 39-year-old man presented to a previous hospital with sudden onset of hematochezia. He underwent emergency lower gastrointestinal endoscopy, which revealed bleeding from the appendiceal orifice. Appendiceal bleeding was suspected, and the patient was referred to our department for surgical management. Contrast-enhanced CT performed at our hospital showed an enlarged appendix and a high-density area in the lumen. In conjunction with the colonoscopy findings, he was diagnosed with appendiceal bleeding and underwent an emergency appendectomy. Histopathological examination revealed a shallow ulcer with a torn mucosal fascia; however, most of the submucosal layer remained intact. Capillaries were observed near the disrupted mucosal fascia. The pathological diagnosis was appendiceal bleeding due to Dieulafoy's lesion. The postoperative course was uneventful, and the patient was discharged on POD 4.

**CONCLUSIONS:**

Symptomatic appendiceal hemorrhage with massive bleeding should be considered for emergency surgical intervention, given the possibility of bleeding due to a Dieulafoy’s lesion.

## Abbreviation

LGIB
: lower gastrointestinal bleeding

## INTRODUCTION

Acute LGIB is common in clinical practice. Colorectal diseases, such as colonic diverticular bleeding, ischemic colitis, and rectal ulcers, are common causes.^1)^ Appendiceal bleeding is an extremely rare but important cause of LGIB.

Herein, we report a case of appendiceal bleeding due to a Dieulafoy’s lesion.

## CASE PRESENTATION

**Fig. 1:**
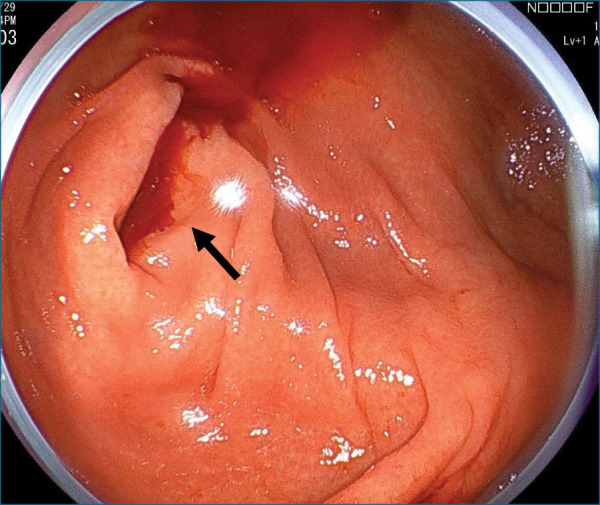
Lower gastrointestinal endoscopy performed at the referring hospital. Active bleeding was observed at the appendiceal orifice (black arrow).

**Fig. 2:**
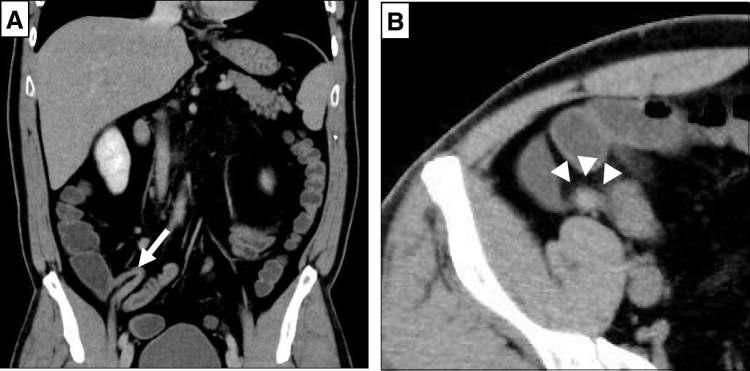
Contrast-enhanced CT. (**A**) The appendix appeared enlarged (white arrow). (**B**) A hyperdense area was identified within the appendiceal lumen (white arrowheads).

**Fig. 3:**
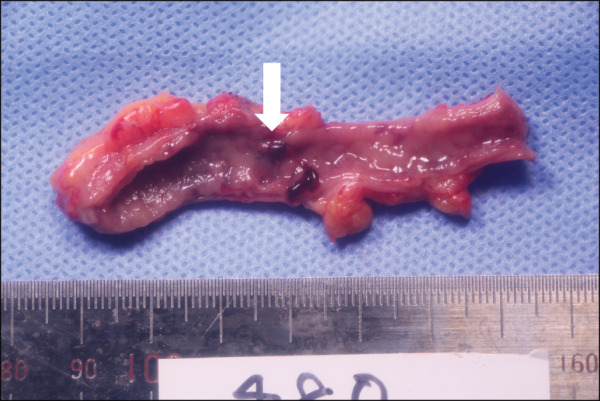
Resected specimen. A blood clot was present within the specimen, but no macroscopic ulceration or signs of diverticulitis were noted. Approximately 35 mm from the oral margin, a small hematoma with exposed capillaries was observed (white arrow).

**Fig. 4:**
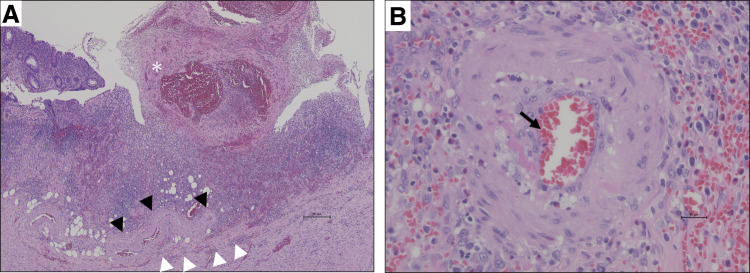
Histopathological findings. (**A**) Hematoxylin–eosin staining (×40). Mucosal erosion is seen adjacent to the hematoma (asterisk). The mucosal fascia (white arrowhead) is partially disrupted, whereas most of the submucosal layer remains preserved. Capillaries are located near the area of mucosal fascial injury (black arrowhead). (**B**) Hematoxylin–eosin staining (×400). The vessel walls show fibrin deposition (black arrow), consistent with vascular injury and subsequent reparative changes.

A 39-year-old man with no medical history presented to our hospital with a sudden onset of hematochezia. Proctoscopy revealed the rectum filled with coagulum, and the patient underwent emergency lower gastrointestinal endoscopy. It showed bleeding from the appendiceal orifice (**Fig. 1**). No diverticula or tumors were observed in the colon. Although temporary hemostasis was achieved with fibrin plugs, surgical management was deemed necessary, and the patient was transferred to our hospital. On arrival, he had no abdominal symptoms, and abdominal examination showed no tenderness or distention. His blood pressure was normal, but tachycardia (109 bpm) was noted. Laboratory testing revealed mild anemia with a hemoglobin level of 11.7 g/dL. Contrast-enhanced CT performed at our hospital showed an enlarged appendix and a hyperdense area in the lumen (**Fig. 2**). Based on the endoscopic and imaging findings, he was diagnosed with appendiceal bleeding and underwent emergency open appendectomy. Intraoperatively, the appendix was enlarged and dark red in color, indicative of hematoma retention. The resected specimen contained a blood clot without any visible ulcers or diverticulitis. A small hematoma and exposed capillaries were observed 35 mm from the oral margin (**Fig. 3**). Pathological examination revealed mucosal erosion around the hematoma (**Fig. 4**). The mucosal surface exhibited small arteries where the mucosal fascia was indistinct, and small clots were present on the surface of the mucosa. Most of the submucosa remained undamaged. The vessel walls were covered with fibrin, suggesting vascular disruption and repair. Histopathological diagnosis revealed appendiceal bleeding due to Dieulafoy’s lesion. He was discharged from our hospital 4 days after surgery without major complications.

## DISCUSSION

We often encounter acute LGIB in daily practice, but most cases are caused by the colon or rectum. The most common etiology is diverticular bleeding, at 64%, followed by ischemic colitis, post-endoscopy bleeding, and rectal ulcers.^1)^ Appendiceal bleeding is extremely rare and can easily be overlooked or misdiagnosed. Xing et al. reported that 6 out of 28175 cases were identified as appendiceal bleeding, accounting for 0.021% of LGIB causes.^2)^ Appendiceal bleeding has been attributed to several causes, including appendicitis,^2)^ diverticulum,^3)^ angiodysplasia,^4)^ Dieulafoy’s lesion,^5)^ and appendiceal ulcer,^6)^ and, in some cases, unknown origins.

Among the reported etiologies of appendiceal bleeding, Dieulafoy’s lesion represents an exceptionally uncommon cause. A Dieulafoy’s lesion is best understood not as a distinct disease entity, but as a pathological vascular abnormality characterized by a persistent large-caliber submucosal artery that becomes exposed through minimal mucosal erosion, leading to significant gastrointestinal bleeding in the absence of ulceration or inflammation. It is estimated to cause 1%–2% of upper gastrointestinal bleeding, but its true frequency is hard to determine because it often shows no symptoms until bleeding occurs, making diagnosis difficult.^7)^ Underrecognition may also explain its apparent rarity.^8)^ While the stomach, particularly along the lesser curvature, is the most frequent site, extragastric occurrences have been documented in the duodenum, colon, and esophagus.^9)^ Involvement of the appendix is exceedingly rare.

A review of previously reported cases of appendiceal Dieulafoy’s lesions^5,10–13)^ (**Table 1**) reveals several common clinical features. In these reports, most patients presented with sudden-onset, painless hematochezia. Hemodynamic instability, including tachycardia or loss of consciousness, was frequently observed despite the absence of inflammatory findings on imaging or intraoperative inspection. Colonoscopy was the primary diagnostic modality in most reports; however, contrast-enhanced CT was also performed in some cases and contributed to the identification of appendiceal intraluminal high-density material suggestive of hemorrhage. Endoscopic identification was often challenging because the bleeding source was located deep within the appendiceal lumen, and appendectomy was ultimately required in all cases. Consequently, definitive diagnosis was typically established only after appendectomy and histopathological confirmation.

**Table 1:** Reported cases of appendiceal bleeding due to Dieulafoy’s lesion

No.	Year	Age/sex	Presentation	Hemodynamic status	Diagnostic modality	Inflammatory findings	Treatment	Rationale for surgery	References
1	2015	68/M	Massive hematochezia	Stable	Colonoscopy	Absent	LaparoscopicAppendectomy	Active bleeding	^5)^
2	2011	75/M	Melena	Loss of consciousness	Colonoscopy + contrast CT	Absent	Appendectomy	Active bleeding	^10)^
3	2020	21/F	Massive hematochezia	Loss of consciousness	Colonoscopy + contrast CT	Absent	LaparoscopicAppendectomy	Hemodynamic instability	^11)^
4	2022	32/M	Massive hematochezia	Loss of consciousness	Colonoscopy + contrast CT	Absent	LaparoscopicAppendectomy	Active bleeding	^12)^
5	2025	26/M	Hematochezia	Stable	Colonoscopy	Absent	LaparoscopicAppendectomy	Diagnostic uncertainty	^13)^
Our case	2025	39/M	Hematochezia	Tachycardia	Colonoscopy + contrast CT	Absent	Appendectomy	Risk of rebleeding	

In the present case, colonoscopy demonstrated bleeding from the appendiceal orifice without other colonic pathology. Although continuous active bleeding was not observed during colonoscopy, the bleeding appeared to cease spontaneously and temporarily. This intermittent pattern made it difficult to confirm definitive hemostasis endoscopically. Contrast-enhanced CT revealed intraluminal hyperdensity within the appendix without surrounding inflammatory changes. Although CT has been reported previously, our case further illustrates that imaging findings may support the suspicion of appendiceal hemorrhage even in the absence of appendicitis.

From a diagnostic standpoint, appendiceal origin should be considered when colonoscopy identifies bleeding from the appendiceal orifice and no alternative source is detected. Imaging may provide supportive findings; however, the absence of inflammatory changes does not exclude clinically significant hemorrhage. Moreover, intermittent bleeding or spontaneous temporary hemostasis may complicate endoscopic evaluation and delay definitive diagnosis.

Based on findings from previously reported cases and the present case, several practical diagnostic clues may be proposed. Appendiceal hemorrhage due to Dieulafoy’s lesion should be suspected when: (1) sudden painless hematochezia occurs without identifiable colonic pathology; (2) bleeding is observed from the appendiceal orifice; and (3) inflammatory findings are minimal despite hemodynamic compromise. Recognition of this clinical pattern may facilitate early surgical consultation and prevent delayed definitive treatment.

Regarding treatment strategy, although endoscopic procedures can provide temporary hemostasis, they may not address the underlying vascular lesion. Given the risk of rebleeding and difficulty in achieving durable endoscopic control, early surgical intervention should be considered in patients with ongoing bleeding or hemodynamic compromise. Appendectomy not only provides definitive hemostasis but also enables pathological confirmation of the diagnosis.

Histopathologically, our case demonstrated mucosal erosion with exposure of a submucosal artery and preservation of the surrounding submucosal layer, consistent with the characteristic features of Dieulafoy’s lesion. The absence of significant inflammation or ulceration further supports the diagnosis and distinguishes it from other causes of appendiceal bleeding.

In conclusion, although appendiceal Dieulafoy’s lesion is extremely rare, it should be considered in the differential diagnosis of unexplained massive LGIB, particularly when bleeding from the appendiceal orifice is identified endoscopically. Recognition of its characteristic clinical pattern—sudden painless hematochezia, minimal inflammatory findings, and potential hemodynamic instability—may facilitate timely surgical management and prevent recurrent bleeding. Accumulation of similar cases may help establish clearer diagnostic strategies for appendiceal hemorrhage in patients with unexplained LGIB.

## CONCLUSIONS

We described a rare case of appendiceal bleeding caused by a Dieulafoy’s lesion. Although extremely uncommon, this condition should be suspected in patients presenting with sudden LGIB when colonoscopy reveals bleeding from the appendiceal orifice without inflammatory findings. Contrast-enhanced CT may provide supportive evidence; however, definitive diagnosis often requires surgical resection. Given the limitations of endoscopic management and the risk of rebleeding, early appendectomy should be considered in cases with ongoing bleeding or hemodynamic instability.
